# The Discovery of an Iridium(III) Dimer Complex as a Potent Antibacterial Agent against Non-Replicating *Mycobacterium smegmatis*

**DOI:** 10.3390/polym10030297

**Published:** 2018-03-09

**Authors:** Guojian Liao, Xixi Peng, Ting Li, Zhengyuan Ye, Xiaohong Xiang, Chen Fu

**Affiliations:** 1 Institute of Modern Biopharmaceuticals, College of Pharmaceutical Sciences, Southwest University, Chongqing 400715, China; gjliao@swu.edu.cn (G.L.); pxxxi15@163.com (X.P.); yzyuan99@163.com (Z.Y.); 2 College of Chemistry and Pharmaceutical Engineering, Nanyang Normal University, Nanyang 473061, Henan, China; 3 School of Pharmacy, Chongqing Medical and Pharmaceutical College, Chongqing 401331, China

**Keywords:** *M. tuberculosis*, iridium dimer complex, bactericidal, ROS, non-replicating

## Abstract

Novel agents are urgently needed to rapidly kill drug-resistant *Mycobacterium tuberculosis*. Noble metal complexes, particularly polypyridyl iridium complexes serving as therapeutic agents, have attracted considerable interest recently, due to their significant cytotoxic or antimicrobial activities. Here, we reported an polypyridyl iridium dimer complex [Ir(ppy)_2_Cl]_2_ (**3**), with ppy = phenylpyridine, which was found to be active against both exponential growing and non-replicating *M. smegmatis*, with minimum inhibitory concentration values of 2 μg/mL, and exhibited rapid bactericidal kinetics, killing pathogens within 30–60 min. Moreover, **3** was demonstrated to generate a large amount of reactive oxygen species and to be effective in drug-resistant strains. Taken together, the selectively active iridium(III) dimer complex showed promise for use as a novel drug candidate for the treatment of *M. tuberculosis* infection.

## 1. Introduction

*Mycobacterium tuberculosis* is an important human pathogen that causes life-threatening infections, claiming around 1.5 million lives each year [1]. With the emergence of multidrug resistance, *M. tuberculosis* poses a serious public health threat [2]. It is estimated that about 450,000 individuals developed the multidrug-resistant tuberculosis (MDR-TB) in 2012, and only fewer than 20% of MDR-TB patients accessed treatment [3]. Therefore, there is a growing unmet medical need to discover novel agents to kill *M. tuberculosis* rapidly, resulting in the fast reduction of the bacterial burden and restriction of the development of drug resistance [4,5,6]. 

Polypyridyl late transition metal complexes haven shown remarkable applications in chemical biology and medicinal chemistry over the last decade [7,8,9]. However, only very recently has there been comprehensive interest in their antimicrobial properties. Noble metal complexes, particularly iridium complexes have been extensively explored as anticancer agents due to their unique modular system, the recognition and binding properties of which can be easily varied by ligand-exchange reactions [10,11,12,13,14]. To the best of our knowledge, only limited iridium complexes have been reported as antibacterial agents to date [15]. Recently, Karpin and co-workers reported that iridium complexes with hydrophobic l-amino acids have antibiotic activity against *Mycobacterium* spp. [16]. However, these reported iridium complexes serving as antimicrobial agents suffer from limitations with respect to their high MIC values, and their possessing bacteriostatic, rather than bactericidal, activity, regardless of the bacterial growth state. We were therefore seeking iridium complexes with new scaffolds that could be employed as potent antibacterial agents against *Mycobacterium tuberculosis*. 

**Figure 1:**
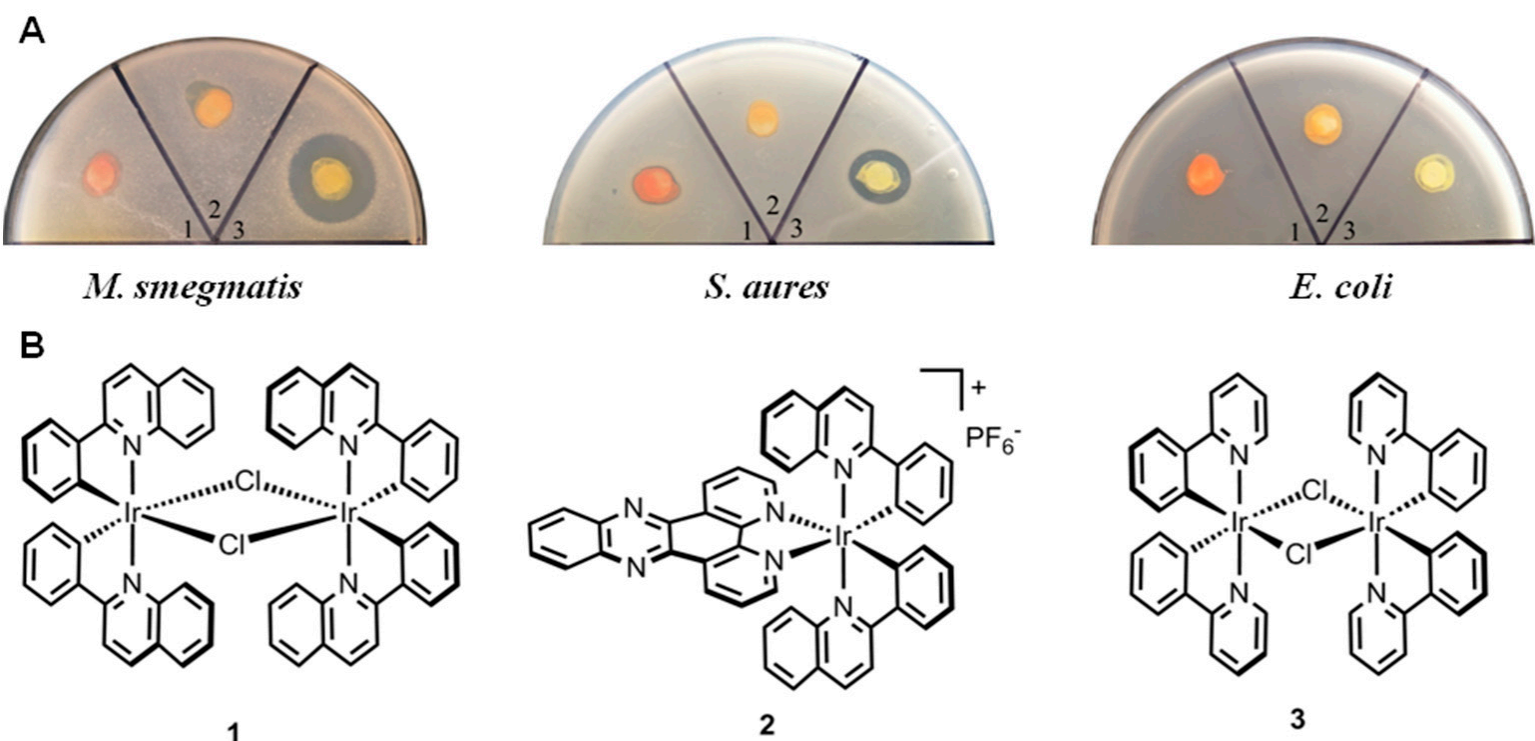
Anti-bacterial activity of complexes **1**–**3** as determined by the disk diffusion assay. (**A**) Strains including *S. aureus* ATCC 33591(MRSA), *E. coli* ATCC25922 and *M. smegmatis* mc^2^ 155; (**B**) Chemical structures of iridium complex **1**–**3**.

A careful examination of the literature directed our focus to the classic polypyridyl iridium(III) dimer complexes [17,18,19]; we here investigated antimicrobial activities of the phenylpyridyl iridium dimer complexes, [Ir(pq)_2_Cl]_2_ (**1**) and[Ir(ppy)_2_Cl]_2_ (**3**), with pq = quinoline and ppy = phenylpyridine, respectively. Both of these complexes have been frequently used as starting materials for the syntheses of heteroleptic iridium(III) polypyridyl complexes. Moreover, the bridging chlorine ligands are labile, similarly to the chlorine in cisplatin, which is a common chemotherapy medication used to treat a number of cancers. In addition, we also studied the antimicrobial activity of [Ir(ppy)_2_(dppz)]_2_ (**2**), with dppz = dipyrido[3,2-a:2′,3′-c]phenazine, which possesses an analogous structure to the classical metallo-intercalator [Ru(phen)_2_(dppz)]^2+^ [20], phen = 1,10-phenanthroline, as shown in Figure 1B. 

*Mycobacterium smegmatis* is a fast-growing and non-pathogenic organism closely related to *M. tuberculosis*. They share vital physiological features, such as dormancy and regulation mechanisms [20]. In this study, we investigated the antibacterial activities of the complexes against drug-susceptible and drug-resistant *M. smegmatis*. The results showed that **3** could be used as a rapidly killing agent, and the bactericidal effect of **3** on *M. smegmatis* was owing to ROS production. The dramatic killing of drug-susceptible and -resistant *M. smegmatis* suggests that cyclometalated iridium dimer complexes may provide a leading structure for the further development of highly potent bacterial agents against *M. tuberculosis*.

## 2. Materials and Methods 

### 2.1. Tested Compounds

The iridium complexes **1** [21], **2** [22] and **3** [23] were synthesized according to the published procedures. ^1^H-NMR spectra were recorded on a Bruker Advance (400 MHz) (Bruker, Karlsruhe, Germany) at ambient temperature, and were consistent with the respective reported literature. Complexs **1**, **2** and **3** were solubilized in dimethyl sulfoxide (DMSO). 2,2′-bipyridyl and thiourea were purchased from Sangon Biotech Co. (Shanghai, China), and norfloxacin was purchased from Sigma-Aldrich (St. Louis, MO, USA).

### 2.2. Strains and Media

*M. smegmatis* mc^2^ 155 was cultured in Middlebrook 7H9 broth (Becton Dickinson, Shanghai, China) supplemented with Tween 80 (0.05% *w*/*v*), glycerol (0.5%) and glucose (0.5%) or was grown on 7H10 agar supplemented with glycerol (1%) and glucose (0.5%). *Staphylococcus aureus* ATCC 33591(MRSA), *S. aureus* ATCC 25923(MSSA), *Escherichia coli* ATCC25922 and *Pseudomonas aeruginosa* PAO1 were grown in Tryptic soy broth (TSB) medium. *Cryptococcus neoformans* H99 and *Candida albicans* ATCC90028 were grown in Yeast Extract Peptone Dextrose (YPD) medium. 

### 2.3. Compound Susceptibility Testing

Sensitivity of bacteria to iridium complexes was measured using the disk diffusion method as described previously [24]. The minimum inhibitory concentration (MIC) of complexes **1**–**3** was determined by broth microdilution methodology as recommended by the clinical and laboratory standards Institute (CLSI) guidelines. Briefly, cultures were incubated in 96-well microtiter plates in the presence of eight two-fold serial dilution of complexes **1**–**3**. Freshly prepared 10^5^ bacterial cells or 10^2^ fungal cells were added and incubated for 24 or 48 h at 37 °C, respectively. The MIC was defined as the lowest concentration of compounds with no visible growth.

### 2.4. Starvation Conditions

The nutrient starvation culture was prepared as described previously [25]. Briefly, exponential phase cultures were pelleted and washed twice with PBS before being resuspended in PBS at 10^7^ Colony-Forming Units (CFU)/mL. Cells were then transferred to standing flasks and incubated at 37 °C with constant rolling at 110 rpm for 10 days. The cultures were then diluted to 10^6^ CFU/mL and treated with 20 μg/mL of **3** or 10 μg/mL (10× MIC) of norfloxacin. In parallel, exponential growth cultures in 7H9 medium was exposed to the same concentration of compounds and the same treatment time to determine the bactericidal effect on replicating cells. Bactericidal activity was determined by CFU enumeration on 7H10 agar.

### 2.5. Measurement of Intracellular Reactive Oxygen Species (ROS)

ROS were measured using the Reactive Oxygen Species Assay Kit (Beyotime Institute of Biotechnology, Shanghai, China) as previously described [26,27]. Briefly, *M. smegmatis* was grown to exponential-phase and cultivated in the presence of indicated concentrations of compounds. Following 2 h incubation, a final concentration of 10 μM DCFH-DA was added to cultures for 20 min at 37 °C. They were then washed twice with 1× PBS and resuspended in PBS. DMSO and norfloxacin were served as controls. Fluorescence was analyzed using a Tecan Infinite 200 PRO microplate reader (Tecan, Shanghai, China).

### 2.6. Protection by Iron Chelator and Hydroxyl Radical Scavenger 

To examine the protection provided by iron chelator and hydroxyl radical scavenger, *M. smegmatis* was prepared as above and treated with 2,2′-bipyridyl (250 μM; 50% MIC) and thiourea (100 mM; 50% MIC) 10 min prior to initiation of antimicrobial treatment. Growth inhibition was determined by measuring the viable cells at the indicated periods of time.

### 2.7. Generation of Norfloxacin-Resistant Mutants

Norfloxacin-resistant strains were obtained by the multistep selection method as described previously [28]. *M. smegmatis* was cultured in the presence of subinhibitory concentration of norfloxacin (1 μg/mL). After 48 h incubation at 37 °C, cells at 10^9^ CFUs were plated on 7H10 medium containing 4 μg/mL of norfloxacin. Colonies of first-step mutant strains were cultured in 7H9 medium without drug and then by plating 10^9^ CFUs containing 8 μg/mL of norfloxacin to generate second-step mutants. This process was repeated, and three mutants were isolated, named N4, N8, N16.

## 3. Results and Discussion

### 3.1. Complex **3** Displays Selective Activity against M. smegmatis

The antibacterial activities of **1**–**3** were investigated by using the Kirby-Bauer disk diffusion assays. Accordingly, gram-positive strain *Staphylococcus aures*, gram-negative strain *E. coli* and *M. smegmatis* were plated on an agar dish, and disks soaked with solution of the complexes **1**–**3** (50 μg). As shown in Figure 1A, **2** did not exhibit activity to any bacteria, which could be attributed to the absence of labile chlorine ligand. Compared to **1**, **3** displayed much more antibacterial activity against *M. smegmatis* and *S. aures*, which suggests that less bulky ligands may be more beneficial for antimicrobial activity. While **3** displayed potent activity against *M. smegmatis*, the minimum inhibitory concentration (MIC) was determined. As shown in Table 1, treatment with **3** inhibited the growth of *M. smegmatis* with a MIC of 2 μg/mL.

Considering that *M. smegmatis* is an established surrogate for screening compounds with inhibitory activity against *M*. *tuberculosis*, and the obvious antibacterial activity of **3** towards *M. smegmatis*, we only focused on the work of **3** in the following study. Accordingly, to further evaluate the antibacterial activity of **3**, it was used to screen activity against a wide range of pathogenic bacteria, such as *E. coli*, *P. aeruginosa*, and *S. aureus* (MRSA or MSSA), and pathogenic fungi such as *C. albicans* and *C. neoformans*. MIC against microbial pathogens was beyond 16 μg/mL (Table 1), demonstrating that **3** exhibited selective activity against *M. smegmatis*. 

### 3.2. Complex **3** Displays Potent Bactericidal Activity against M. smegmatis

**Figure 2:**
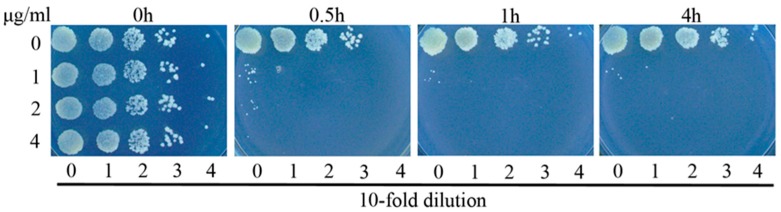
Complex **3** is bactericidal against *M. smegmatis*. Bacterial cells were inoculated in 7H9 medium, and cultured either without drug or in the presence of **3** at various concentrations. At the indicated time points, aliquots of cell suspension were transferred and plated on drug-free 7H9 medium CFU after 24 more hours of incubation.

To further explore the activities of complex **3** against *M. smegmatis*, we performed time killing experiments. Complex **3** displayed potent bactericidal activity against *M. smegmatis*, resulting in a 3-log reduction in viable cells 0.5 h of treatment with compound concentrations of 2 μg/mL (Figure 2). No viable cells were observed at 1h at 2 μg/mL. On the basis of these results, it can be concluded that **3** is a fast killing agent with great potential.

### 3.3. Complex **3** Displays Activity against Non-Replicating M. smegmatis

**Figure 3:**
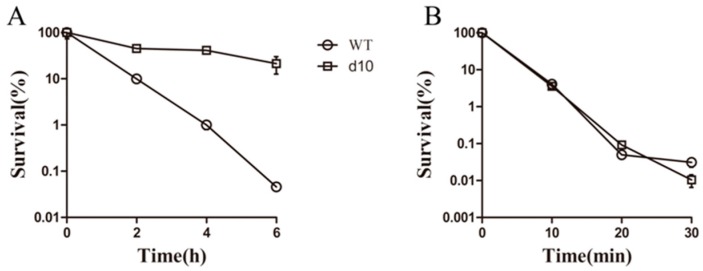
Complex **3** displays potent activity against non-replicating mycobacteria. Ten-days-starved and exponential-phase *M. smegmatis* cultures were treated in triplicate with norfloxacin (**A**) or **3** (**B**) at several time points. Cultures were washed twice with PBS and their viability assessed by plating followed by CFU counting. Values represent the means ± standard errors of triplicate determinations.

It has been reported that most antitubercular drugs exhibit reduced bactericidal activities against non-replicating starved bacilli, contributing to latent infection. Nutrient-deprived *M. smegmatis* is one of the established models for studying non-replicating starved states. Exposure to 20 μg/mL (10× MIC) **3** led to a 3-log CFU decrease under both culture conditions (Figure 3B). **3** killed non-replicating bacteria and showed equal efficacy against rapidly growing cells. The killing efficacy of **3** was greater that of norfloxacin (Figure 3A), which is known to retain bactericidal activity under non-replicating states, although it is significantly less active against non-replicating than against exponential-phase cells. These results demonstrated that **3** exhibited potent activity against non-replicating *M. smegmatis* and may target processes that are essential for survival even under non-replicating conditions. 

### 3.4. Complex **3** Was Active against Norfloxacin-Resistant Strains

**Figure 4:**
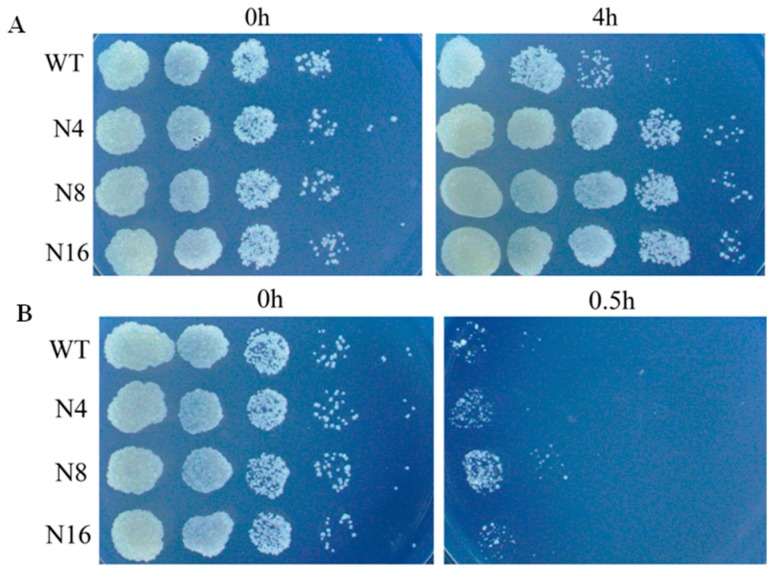
Complex **3** shows activity against norfloxacin-resistant strains. (**A**) Laboratory-generated mutants (N4, N8 and N16) are resistant to norfloxacicn; (**B**) WT and norfloxacin-resistant strains are susceptible to **3**. The MIC of WT against norfloxacin is 2 μg/mL, the MIC of resistant strains is 16–32 μg/mL.

One of the main obstacles to TB eradication is the high prevalence of drug-resistant strains. To assess of the effect of **3** on drug-resistant strains, norfloxacin-resistant strains were treated with **3**. Laboratory-generated resistant strains were obtained by spontaneous mutation under different concentrations of norfloxacin. Three resistant strains with MICs 8 to 16 folds greater than that of WT strains were isolated (N4, N8 and N16) (Figure 4A). **3** displayed similar efficacy against norfloxacin-resistant strains, resulting in a 100-fold to 1000-fold decrease in CFU in 0.5 h treatment (Figure 4B). The lack of cross-resistance with currently used drug class suggested that **3** may retain activity against drug-resistant strains and may have novel modes of action.

### 3.5. Antibacterial Mechanism of Complex **3**

**Figure 5:**
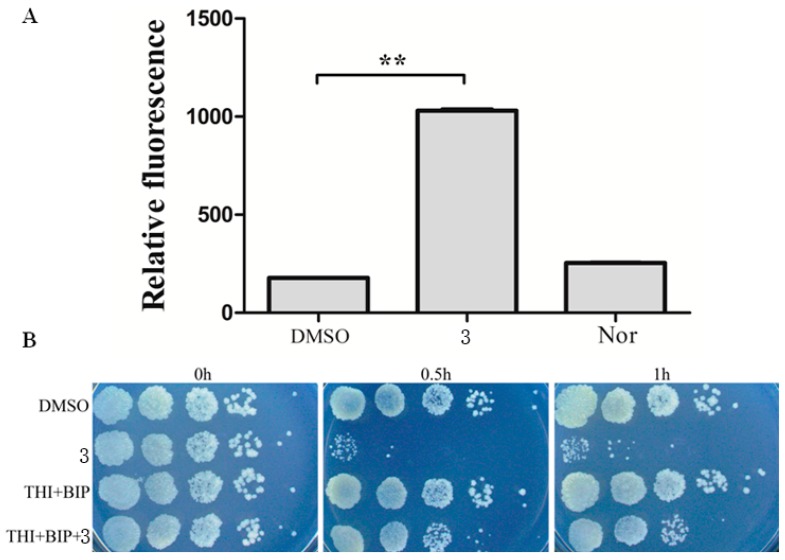
Complex **3** triggers endogenous ROS production in *M. smegmatis*. (**A**) Percentages of intracellular increase in ROS generation in the presence of 10 times of MIC of **3**. Norfloxacin represents the positive control for ROS production. Data are shown as mean ± SD of triplicate wells. ** *p* < 0.01; (**B**) Effects of a ferrous chelator and a hydroxyl radical scavenger on **3** lethality. Exponentially growing *M. smegmatis* cells were preincubated with 250 μM biphyridyl and 100 mM thioura for 10 min before they were treated with 10 times of MIC of **3** for 0.5 or 1 h. At least three replicate experiments were performed, and each had similar results.

The mechanism of Iridium(III) complexes killing bacterial pathogens remains largely unknown. These complexes can bind DNA and RNA, interact with cell wall, as well as generate ROS. Given that **3** is a rapid killing agent and is redox-active, it is likely to generate ROS via electron transfer to oxygen, as previously reported. We examined the induction of cellular oxidative stress by **3** (1 μg/mL) on *M. smegmatis* compared with a negative control (DMSO). It could be observed that **3** did indeed induce strong oxidative stress (*p* < 0.01), and the oxidative stress induced by **3** was stronger than that of norfloxacin, which is known to exert its antimicrobial activity by inducing cellular oxidative stress on bacteria (Figure 5A).

To further examine the role of ROS generation in complex **3**-mediated killing, iron chelator Biphyridyl and radical scavenger thiourea, which are able to alleviate the effect of ROS on cell viability, were added to the culture in the presence of compound concentration of 1 μg/mL. Cotreatment with sub-inhibitory concentrations of biphyridyl and thiourea did not affect the growth of *S. smegmatis*. However, the same cotreatments reduced **3**-mediated killing, resulting in 10 and 100 fold reduction of efficacy after 0.5 and 1 h treatment, respectively (Figure 5B). Taken together, these results suggested the involvement of ROS in the **3**-mediated lethality.

## 4. Conclusions

In summary, we here reported that a polypyridyl iridium dimer complex **3** displayed potent and selective activity against *M. smegmatis*. Furthermore, **3** retained activity against laboratory-generated norfloxacin-resistant strain of *M. smegmatis*. Mode of action studies indicated that the antimicrobial activity of **3** was potentially due to the production of ROS. On the basis of our findings, it can be concluded that cyclometalated iridium dimer complexes may provide a leading structure for the further development of highly potent bacterial agents against *M. tuberculosis* infection.

## Figures and Tables

**Table 1:** Activity of **3** against pathogenic microorganisms.

Organism and Genotype	MIC (μg/mL)
*M. smegmatis*	2
*S. aureus* (MSSA)	16
*S. aureus* (MRSA)	32
*P. aeruginosa*	>64
*E. coli*	>64
*C. albicans*	>64
*C. neoformans*	>64
